# Exploring public views on the use of synthetic datasets for research - results from the DELIMIT study

**DOI:** 10.23889/ijpds.v9i5.2919

**Published:** 2024-09-18

**Authors:** Fiona Lugg-Widger, Claire Nollett, Lucy Brookes-Howell, Mike Robling, Rob Trubey

**Affiliations:** 1 Centre for Trials Research, Cardiff University

## Background

Synthetic data refers to datasets derived from ‘real’ administrative data records but sufficiently abstracted to minimise disclosure risk. They can be an efficient way for researchers to learn about the properties of datasets and inform data requests, prior to data applications.

However, little is understood about how the UK public feel about the creation and use of such datasets for research purposes.

## Methods

We worked with a community engagement agency (Egality Health) to recruit a diverse UK public panel, to discuss issues relating to the use of synthetic data in research.

Across four workshops, participants listened to presentations from topic experts, and deliberated on perceived benefits and risks of synthetic data, ethical issues and reflections on how to communicate clearly about synthetic data. Members agreed a set of recommendations for researchers, data custodians and policy makers.

## Results

We will summarise key themes and recommendations from the workshops, explaining how these might help inform future policy.

We will reflect on how these results fit with parallel work that has explored the views of researchers and data custodians on the use of synthetic data for research, and discuss potential international implications of our findings.

## Conclusions and Implications

As with any research that seeks to draw on the public’s data, it is vital that researchers and policy-makers engage with and involve the public in any decision making around synthetic data.

In presenting findings from this project, we seek to advocate for a public voice in this topic and to ensure that future policy - both in the UK and internationally - is aligned with public attitudes.

